# From Vine to Sparkle: An Analytical and Sensory Evaluation of Sparkling Wines from Some Romanian Native Grapes

**DOI:** 10.3390/foods15020353

**Published:** 2026-01-18

**Authors:** Dragoș-Florin Popa-Grosaru, Bettina-Cristina Buican, Camelia Elena Luchian, Lucia Cintia Colibaba, Elena Cristina Scutarașu, Marius Niculaua, Constantin Bogdan Nechita, George Ștefan Coman, Elena Cornelia Focea, Tiberiu Andrieș, Diana Ionela Popescu (Stegarus), Valeriu V. Cotea

**Affiliations:** 1 “Ion Ionescu de la Brad” Iași University of Life Sciences, 3rd M. Sadoveanu Alley, 700490 Iași, Romania; dgrosaru@gmail.com (D.-F.P.-G.); bettina.buican@iuls.ro (B.-C.B.); cintia.colibaba@iuls.ro (L.C.C.); cristina.scutarasu@iuls.ro (E.C.S.); george.coman@iuls.ro (G.Ș.C.); tarcancornelia2@gmail.com (E.C.F.); andries.tiberiu@iuls.ro (T.A.); valeriu.cotea@iuls.ro (V.V.C.); 2 Romanian Academy–Iași Branch, Research Center for Oenology, 9H, M. Sadoveanu Street, 700490 Iași, Romania; niculaua@acadiasi.ro (M.N.); bogdan.nechita@acadiasi.ro (C.B.N.); 3 National Research and Development Institute for Cryogenic and Isotopic Technologies, ICSI Ramnicu Valcea, 4th Uzinei Street, 240050 Ramnicu Valcea, Romania; diana.stegarus@icsi.ro

**Keywords:** oenology, traditional method, volatile compounds, lees, sensory profile

## Abstract

The increasing global demand for sparkling wines has encouraged the exploration of alternative grape varieties and emerging production regions. This study evaluated the potential of three indigenous Romanian grape varieties (Fetească regală, Tămâioasă românească, and Fetească albă) for sparkling wine production using the méthode champenoise, with grapes sourced from the ullu Mare region. The wines were characterized at two aging intervals (9 and 36 months on lees), with analyses performed on both disgorged and undisgorged samples to assess changes in physicochemical parameters, color attributes, volatile composition, and sensory properties. All varieties exhibited relatively high acidity (6.12–6.53 g/L), particularly Fetească regală (6.37–6.53 g/L), supporting their suitability for sparkling wine production. Extended lees aging enhanced the development of complex tertiary and quaternary aromas while preserving intrinsic floral and fruity attributes. Volatile analysis revealed aging-related increases in higher alcohols and medium-chain fatty acids, with 1-pentanol reaching 106.8 mg L^−1^ and octanoic acid increasing from approximately 4.2 to 7.9 mg L^−1^ after 36 months. Principal component analysis explained over 70% of the total variance, discriminating wines according to grape variety and maturation time. This study aimed to provide a detailed characterization of these sparkling wines, integrating physicochemical, chromatic, volatile, and sensorial analyses to evaluate their quality and enological potential.

## 1. Introduction

Sparkling wine has a long-standing history and continues to enjoy widespread popularity worldwide. The distinctive terroirs and grape varieties of various wine-producing regions are reflected in its vast diversification, refinement, and regional adaptation over the centuries. Since the 17th century, wines made with the traditional bottle-fermentation method, known as *méthode champenoise*, have gained more and more recognition on a global scale [1]. France’s Champagne, Crémant from the other wine regions of France, Franciacorta in Italy, Sekt in Germany, and Cava in Spain are all famous examples [2]. Because of their careful secondary fermentation and lees contact, these sparkling wines stand out for their delicate effervescence, nuanced aroma profiles, and long aging potential. Both historical prestige and shifting consumer tastes for wines that blend elegance, complexity, and sensory sophistication are factors in their widespread appeal [3].

The *méthode champenoise* or classic method is the most famous technique for producing high-end sparkling wines like Cava and Champagne [4,5]. To begin a secondary fermentation that takes place inside the bottle a still base wine is combined with a liqueur de tirage which is a mixture of wine sugar yeasts and nutrients [6,7]. The second fermentation phase is a process that enhances the wine’s overall sensory profile texture and aromatic complexity by allowing it to develop its natural effervescence and aging on lees for a considerable amount of time, typically at least nine months. Although the method is costly and time-consuming, it yields sparkling wines with exceptional elegance, depth and aging potential [8,9].

Romania is home to several distinctive native grape varieties that show promising similarities to the cultivars traditionally used for producing sparkling wines by the *méthode champenoise* [10,11,12]. Although the country is best known for its white grape varieties cultivated in the cooler northern regions and red varieties grown in the warmer southern areas, recent studies have demonstrated that many of these native cultivars possess naturally high acidity and well-balanced compositional profiles [13,14]. These characteristics, which are strongly influenced by terroir and vineyard management, contribute to their potential suitability for sparkling wine base production. The preserved acidity, combined with their delicate aromatic complexity, provides the freshness, precision, and structural balance essential for crafting high-quality, traditionally made sparkling wines with strong aging potential [14,15,16].

**Figure 1:**
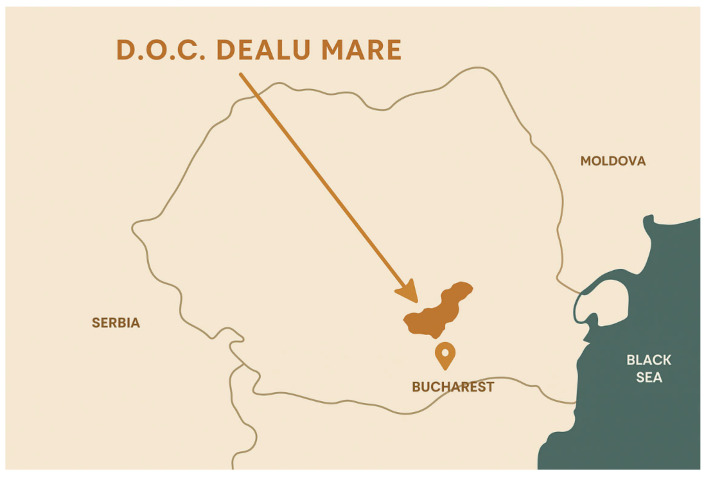
Map of the Dealu Mare Viticultural Region.

With approximately 15,000 hectares of vineyards spread across nine wine centers, including Boldești, Valea Călugărească, and Tohani, Dealu Mare is one of Romania’s most important viticultural regions (Figure 1). Its moderate Mediterranean-influenced climate and mean annual temperature of 10.8–11.2 °C make it the perfect place to grow high-quality grapes, and its terroir and vineyard practices yield grapes with delicate aromatic complexity and preserved acidity, which make them ideal as a base for traditionally produced sparkling wines [17,18].

This study aims to assess the potential of autochthonous grape varieties from the Dealu Mare region, focusing on their suitability for traditionally made sparkling wines. The varieties Fetească albă, Fetească regală, and Tămâioasă românească were selected for their unique organoleptic characteristics, including distinctive floral and fruity aromas, balanced acidity, and structural complexity, which set them apart from common international cultivars. Their long-standing cultivation in the region has also led to strong adaptation to local climatic and soil conditions, making them resilient and reliable for viticultural practices. By evaluating these varieties, the study seeks to demonstrate their potential not only for producing high-quality sparkling wines but also for reinforcing sustainable use of native grape resources and preserving Romania’s viticultural heritage.

## 2. Materials and Methods

### 2.1. Wine Samples

The study was conducted on three different *Vitis vinifera* L. grape varieties, namely Fetească regală (FR), Tămâioasă românească (TR) and Fetească albă (FA) (Table 1), sourced from Dealu Mare wine region, area of Săhăteni–Gura Vadului (45°02′45.2″ N 26°27′24.4″ E). The hand-picking of the grapes was carried out over different years: 2019 and 2021. Harvesting was performed under good sanitary conditions, with grapes collected in approximately 20 kg cases, followed by pressing with careful monitoring of must pH. Settling was performed at 12 °C, using ENDOZYM^®^ ICS 10 Éclair (AEB Group, Italy) clarifying enzymes. The alcoholic fermentation was carried out at a temperature of 14 °C, with 15 g/hL commercial yeast (IOC 18-2007™, Laffort SUPERSTART™ Blanc - Laffort, Bordeaux, France). During maturation, wines underwent successive rackings according to their development.

**Figure 2:**
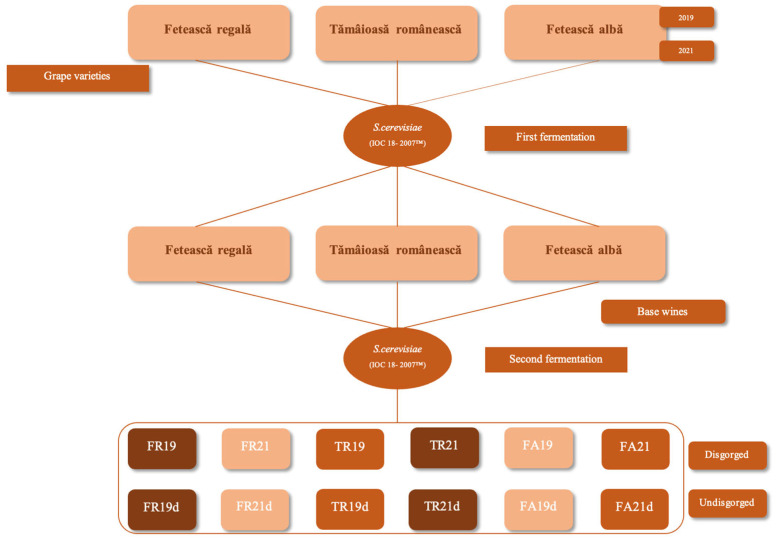
Flow chart of the experimental design. Acronyms used in this table: *S. cerevisiae*—*Saccharomices cerevisiae*, FR—Fetească regală, FA—Fetească albă, TR—Tămâioasă românească.

Prior to the second fermentation in bottles, the wine temperature was raised to 15 °C. The tirage process involved preparing a yeast starter using IOC 18-2007™ at 20 g/hL and Go-Ferm™ (Lallemand Inc., Montreal, QC, Canada) activator at 10 g/hL. A liquor composed of rectified concentrated must, raw material wine was prepared separately, to which the rehydrated yeast solution was added. After homogenization, the wines were bottled in 0.75 L Grand Cru glass bottles. The secondary fermentation lasted between 60 and 90 days post-bottling. Following a period of maturation (9 and 36 months), bottles were disgorged, expedition liquor was added, and the bottles were corked. For analytical purposes, one sample per variety, per year, was retained as an undisgorged sparkling wine. A depiction of the experimental design is provided in Figure 2.

### 2.2. Physico-Chemical Parameters

The main physico-chemical analysis were performed based on accredited methods, according to International Organization of Vine and Wine (OIV), Compendium for interntional Methods of Analysis of Wines and Musts [19]. Alcohol content (% *v*/*v*) was determined by distillation and densimetric measurement (OIV-MA-AS312-01). Volatile acidity (g acetic acid L^−1^) was measured by steam distillation (OIV-MA-AS313-02), while titratable acidity (g tartaric acid L^−1^) was determined by acid–base titration (OIV-MA-AS313-01). Residual sugars (g glucose L^−1^) were quantified using enzymatic methods (OIV-MA-AS311-01). pH was measured using a calibrated pH meter (OIV-MA-AS313-15), and density was determined by densimetric analysis (OIV-MA-AS2-01). Free and total sulfur dioxide (mg L^−1^) were determined by iodometric titration (OIV-MA-AS323-04).

### 2.3. Chromatic Parameters

Chromatic parameters (L*, a*, and b*) were calculated in the CIE Lab* color space using the methodology established by the International Commission on Illumination (CIE) [20]. The chromatic parameters (L*, a*, b*, $C_{ab}^{*}$), color intensity (I), and hue (N) were determined by processing the absorption spectra for each sample using a program called VINCOLOR that was created by the research team [21,22]. Using an Analytik Jena Specord 200 UV-VIS spectrophotometer connected to an IBM-PC and 1 cm optical path glass cuvettes, the chromatic parameters were computed based on the absorption spectra recorded in the visible range (VIS), at a wavelength between 380 and 780 nm. The color difference can be calculated using mathematical models that have been converted into computer programs. Following the color pattern, these programs enable the dissection of any color difference into its constituent hue, lightness, and intensity [20].

### 2.4. Volatile Substances

Volatile compounds were analyzed using a Shimadzu GC-2010 system coupled to a QP2010plus (Shimadzu Corporation, Kyoto, Japan) mass spectrometer. Concentration of volatile analytes was performed with a Shimadzu AOC-5000 auto-injector in ITEX configuration. Samples were first cooled to 4 °C and subsequently degassed in an ultrasonic bath (48 kHz, 10 W) for 90–120 s, using 1 s active pulses followed by 10 s pauses to prevent excessive foaming in 100 mL Erlenmeyer flasks. For headspace preparation, 4 g NaCl and 10 mL of degassed sample were transferred into 20 mL headspace vials sealed with silicone–PTFE septa, followed by the addition of an internal standard (4-methyl-pentan-2-ol, 50 mg/L in absolute ethanol). Samples were equilibrated at 30 °C for 10 min, after which 25 extraction cycles were carried out using a modified 5 mL headspace syringe and a TENAX trap (60/80 mesh), maintained at 50 °C, with a 20 µL/s extraction flow; syringe temperature was held at 40 °C. Analytes retained on the trap were thermally desorbed into a split injector at 260 °C, increasing trap temperature to 250 °C, and 1500 µL of vapor were injected with a split ratio of 10:1. Chromatographic separation was achieved on a Thermo TR-WAXMS column (60 m × 0.25 mm × 0.25 µm) with helium (grade 6.0) at a constant linear velocity of 35.2 cm/min. The temperature program was: 40 °C for 360 s; ramp 4 °C/min to 80 °C (61 s hold); 7 °C/min to 200 °C (30 s hold); 13 °C/min to 250 °C (570 s hold). The MS detector operated at 1.12 kV, with an interface temperature of 250 °C and a quadrupole temperature of 200 °C. Spectra were acquired at 2000 amu/s over 50–300 Da. Compound identification was based on spectral matching (≥75% similarity) against NIST14, Wiley07, and FFNSC 1.2 libraries, while semi-quantitative evaluation was performed relative to the internal standard [14].

### 2.5. Sensory Analysis

The sensory analysis of the experimental samples was performed following the OIV recommendations [23]. Tasting sessions were conducted during the first part of the day to ensure optimal perceptual sensitivity to the targeted descriptors. All samples were assessed at a controlled temperature of 10–12 °C. The sensory profile was characterized by a trained panel from the Iasi University of Life Sciences, composed of 20 evaluators (11 men and 9 women). The assessment focused on key descriptors relevant to sparkling wines, including vegetal nuances, overall fruitiness, and elderflower aroma. Fruitiness was further subdivided into melon, apple, peach, and banana notes. Additional sensory parameters: (persistence, texture, bitterness, sweetness, acidity, yeast-derived aromas, and toasted notes) were also examined. Each attribute was quantified using a 10-point intensity scale, ranging from 0 (not perceptible) to 9 (maximum intensity). Results were compiled, and the arithmetic mean for each descriptor was subsequently calculated.

### 2.6. Statistical Analysis

Data were expressed as the mean and standard deviation values and analyzed using XLSTAT–Basic XLSTAT–Basic, student-type user software, a statistical and data analysis solution (Lumivero, Denver, CO, USA), was used. Prior to hypothesis testing, exploratory data analysis was conducted to assess data distribution and detect potential outliers. Descriptive statistics (mean, standard deviation, minimum, maximum, and quartiles) were computed using XLSTAT–Basic (Lumivero, Denver, CO, USA). Histograms and boxplots were used to evaluate distribution patterns, while outliers were removed only when attributable to recording errors. Normality was assessed using the Shapiro–Wilk test to guide the choice of parametric or nonparametric tests. To explore multivariate structure and reduce dimensionality, Principal Component Analysis (PCA) was performed on standardized variables using the correlation matrix. Component retention followed the Kaiser criterion (eigenvalues > 1), scree plot inspection, and cumulative explained variance (>70%). Factor loadings, correlation circles, and biplots were examined to interpret variable contributions, and Varimax rotation was applied to enhance interpretability. All analyses were conducted at a 95% confidence level, with exact *p*-values reported. Graphical outputs were exported at high resolution, and all analysis workflows were documented to ensure reproducibility.

## 3. Results and Discussion

### 3.1. Physicochemical Parameters

Successful completion of the AF was achieved under all conditions, as illustrated (Table 2). Nevertheless, significant differences emerged among the various experimental setups. Physico-chemical analyses were carried out, encompassing measurements of alcohol content, total acidity, reducing sugars, pH, volatile acidity, free and total sulfur dioxide, and bottle pressure. Alcohol concentrations ranged from 13.03% *v*/*v* to 13.53% *v*/*v*, aligning with the typical values observed in sparkling wines produced using the traditional method [24,25,26]. Similar alcohol contents have been reported for sparkling wines produced internationally using different yeast strains, such as *Saccharomyces cerevisiae* and *Torulaspora delbrueckii*, even when applied in co-inoculation, alcohol levels remained comparable. Furthermore, studies conducted in Argentina on autochthonous grape varieties have reported alcohol contents reaching up to 13.7% (*v*/*v*), in agreement with the values observed in the present study.

The slight variation in alcohol levels among the samples (less than 0.5% *v*/*v*) indicates stable fermentation performance and consistent dosage handling. The highest values were recorded for TR19 (13.53% *v*/*v*) and TR21d (13.42% *v*/*v*). These differences likely stem from the initial sugar content of the must and the overall fermentation efficiency. Despite this modest spread, all samples fall well within a desirable oenological range, supporting an appropriate balance between structure and freshness.

Total acidity, an essential contributor to the freshness and overall structural balance of sparkling wines, ranged from 6.00 to 6.53 g L^−1^ across the samples. The largest differences were linked to the harvest year: wines from 2021 displayed higher acidity, whereas those from 2019 showed the lowest values. Interestingly, the Fetească regală samples reflected this pattern most clearly, registering both the minimum (6 g L^−1^) and maximum (6.53 g L^−1^) values across the two vintages. These shifts may be associated with climatic variations between years, consistent with findings from research conducted in the Dealu Mare region, which reported annual decreases in total acidity ranging from 0.02 to 0.10 g L^−1^ [27]. Despite these fluctuations, the overall range remains narrow, indicating effective control of acidity throughout the winemaking process. The pH values, ranging from 3.03 to 3.39, align with the acidity measurements and fall largely within the optimal interval for sparkling wines, generally considered to be around 3.0–3.3. The slightly higher pH observed in FA21 (3.39) and FA21d (3.32) may have implications for aging capacity and microbial stability, although these values still fall within acceptable limits for traditional-method sparkling wine production. Though Dealu Mare is not conventionally seen as suitable for sparkling wine production, the results highlight its emerging sustainable potential and support the formulation of strategies aimed at optimizing its use for high-quality sparkling wines.

Residual sugar levels ranged from 1.85 to 3.67 g L^−1^, consistent with the Brut category of sparkling wines [28]. The lowest concentrations were found in FA19d (1.85 g L^−1^) and FR21 (1.95 g L^−1^), which corresponded with their relatively higher acidity. Conversely, the highest values, 3.67 g L^−1^ in TR21d and 3.43 g L^−1^ in TR21, were associated with samples showing some of the lower acidity levels. Although these fluctuations are modest, they can subtly affect the perception of sweetness and the overall balance with acidity, leading to slight differences in the sensory impression among the samples [29].

Volatile acidity levels were well below the legal threshold of 1.2 g L^−1^ [19] n all samples, falling within a narrow range of 0.36 to 0.42 g L^−1^. Such values reflect sound fermentation practices and good microbial stability, suggesting a very low likelihood of developing spoilage-related off-flavors [30]. During the second fermentation, or prise de mousse, the yeast consumes the added sugar and releases CO_2_, which dissolves into the wine and results in a supersaturated system [31]. The recorded pressures ranged from 6.2 to 7.4 bar, values that indicate a well-conducted secondary fermentation with effective CO_2_ retention, as sparkling wines generally target pressures above 6 bar. The slightly elevated pressures measured in FR21d, TR21d, FA19d and FA21d (7.4 bar) may contribute to a livelier effervescence and more persistent foam during tasting [32]. These pressure values are consistent with those reported by Cisilotto et al. (2023) [26], who compared traditional and Charmat methods in sparkling wines produced at Chandon (Brazil). All sparkling wine samples complied with the essential physico-chemical criteria for traditional-method production, reflecting reliable fermentation and well-controlled composition. Slight differences in acidity, residual sugar, pH, and pressure indicate potential nuances in sensory perception, yet overall quality and winemaking precision remained consistently high across all samples.

### 3.2. Color Chracterisation

The chromatic parameters, L* (lightness or psychometric clarity), a* (red–green coordinate), b* (yellow–blue coordinate), and C* (chromaticity or saturation), were determined using the CIE L*a*b* 1976 method, based on the recorded absorption spectrum of each wine. The resulting values are presented in Table 3. In addition to these parameters, two further variables were calculated: E* (representing the perceptual color difference) [10] and H* (representing the difference in hue between two colors), using the control sample as the reference.

The chromatic analysis of the sparkling wine samples revealed L* values ranging from 98 to 99.1, indicating a consistently high level of brightness and clarity across all wines. Such values are in agreement with recent studies on traditional-method sparkling wines, where L* values for young or recently bottled wines typically fall in the high 90 [24,33,34]. The chromatic analysis of the sparkling wine samples revealed a coherent and narrowly distributed color profile. The a* values, which ranged from 1.11 to −0.53, point to a subtle greenish tint typical of young sparkling wines with limited oxidative development. Similar tendencies toward slightly negative a* coordinates have been noted in studies of both traditional-method cuvées and tank-fermented sparkling wines, where early-stage wines often retain a faint green hue associated with grape variety and minimal phenolic extraction [35]. This finding is consistent with traditional method for sparkling wines, as reported by Just-Borràs et al. (2024) [24], who found no significant a* difference between ancestral and traditional-method cuvées. Meanwhile, our b* values (4.27–8.04) indicate a pale yellow tone, which aligns with their observation that traditional-method wines are less yellow than ancestral styles [36]. The chroma (C*) values in our samples, ranging from 4.3 to 8.12, fall into the low-to-moderate saturation bracket, comparable to the delicate chroma levels documented in wine color studies. It is important to note that small variations in instrumental settings, such as data interval or scan speed, can influence the measured a* and C* values, indicating that part of the observed variability may reflect measurement conditions rather than true compositional differences. Similarly, common winemaking practices such as fining and stabilization are unlikely to significantly alter a*, supporting the consistency and plausibility of slightly negative a* values across production [37,38].

### 3.3. Volatile Compounds

A total of 36 volatile constituents were identified and measured. These include three aldehydes, seven alcohols, and seventeen esters, alongside three fatty acids, seven terpenes and related terpenoids, as well as two furan-derived compounds (Table 4 and Table 5). Together, they represent the major chemical families typically associated with aroma formation, each contributing distinct sensory notes and biochemical signatures to the overall profile.

#### 3.3.1. Esters

The quantitative assessment of ethyl esters and acetate esters provides essential mechanistic insight into the aromatic diversity and fermentative signature of sparkling wines, as these compounds are widely recognized as major contributors to fruity and floral sensory attributes [39,40]. Their formation is primarily driven by yeast enzymatic pathways during alcoholic and secondary fermentations, although their final concentrations also reflect subsequent chemical transformations, including esterification, hydrolysis, and interactions with lees during maturation [41]. Across the dataset, compounds such as ethyl acetate, ethyl lactate, ethyl caprylate, and ethyl caprate consistently appeared at relatively high concentrations, underscoring their central role in shaping the chemical identity and sensory expression of these wines.

The ester composition showed clear, interrelated influences of grape variety, lees-aging duration, and disgorging, despite all samples being produced with the same yeast strain. The 2019 wines, aged for 36 months on lees, consistently exhibited higher concentrations of the main fruity esters compared to the 2021 wines, which underwent only 9 months of lees contact. Ethyl acetate reached 88 mg L^−1^ in FR19, exceeding all 2021 samples, while ethyl hexanoate was similarly elevated in FR19 (61.75 mg L^−1^) and TR19d (55 mg L^−1^). At moderate concentrations (50–80 mg L^−1^), ethyl acetate imparts pleasant pear- and pineapple-like aromas, but levels above 150 mg L^−1^ may introduce solvent- or glue-like off-notes [37]. All wines in this study remained within the acceptable sensory threshold, although the concentration in FR19 (88.42 mg L^−1^) approached the upper range of positive contribution, indicating a particularly vigorous fermentation. These trends correspond with the well-known impact of extended lees contact, during which yeast autolysis and slow esterification processes gradually enrich the volatile profile of traditional-method sparkling wines [42].

Varietal origin further shaped the ester landscape. Fetească regală wines showed the highest acetate-ester concentrations (59.98–88.42 mg L^−1^), whereas Fetească albă samples were distinguished by increased levels of short-chain ethyl esters, especially ethyl isobutyrate (0.66–1.27 mg L^−1^). A similar pattern was observed for Tămâioasă românească from 2019 (0.94–1.29 mg L^−1^). Disgorging led to moderate but consistent decreases in several esters, particularly the more volatile ones. For example, ethyl acetate declined from 88 mg L^−1^ in FR19 to 67.97 mg L^−1^ in FR19d. Comparable reductions were noted for FA19 and TR21, whereas other samples displayed the opposite tendency. Despite these losses, the disgorged 2019 wines retained substantially richer ester profiles than any 2021 sample, confirming that lees-aging duration was the primary determinant of ester development, followed by varietal differences. Medium-chain ethyl esters, ethyl hexanoate (apple, anise), ethyl caprylate (apple, waxy), and ethyl caprate (soapy, fruity), play a central role in shaping the wines’ fruity character, and their combined abundance is a reliable indicator of fermentation performance. The FR and FA groups contained consistently strong levels of these compounds. FR21d, for instance, showed very high ethyl caprylate concentrations (69.76 mg L^−1^) and ethyl caprate (14.08 mg L^−1^), contributing intense sweet- and tropical-fruit notes. The FA samples also presented robust ester production, particularly FA21, which contained 90.35 mg L^−1^ of ethyl caprylate. These patterns are consistent with fermentations characterized by low levels of stress-induced medium-chain fatty acids and efficient conversion into their corresponding esters. The Tămâioasă românească group exhibited greater variability. TR21 contained particularly high concentrations of ethyl caprylate (93.03 mg L^−1^) and ethyl hexanoate (80.64 mg L^−1^), whereas TR19 showed considerably lower levels. Moreover, TR21 and TR21d displayed exceptionally high isoamyl acetate (banana, pear-candy aromas) at 9.98 and 10.98 mg L^−1^, while TR19 was notable for its elevated ethyl isovalerate (5.37 mg L^−1^). Diethyl succinate was detected across all wines (0.92–1.88 mg L^−1^).

The results obtained in this study are consistent with existing research on traditional-method sparkling wines. Several authors have demonstrated that prolonged lees aging promotes a marked increase in medium-chain ethyl esters due to yeast autolysis and the gradual release of fatty-acid precursors. For instance, Riu-Aumatell et al. (2006) observed a clear rise in compounds such as ethyl hexanoate and ethyl octanoate during extended Cava maturation, in agreement with the elevated ethyl hexanoate and ethyl caprylate detected in the 36-month samples of the present work [43]. Recent investigations, on Champagne also corroborate this trend, reported substantial increases in acetate esters after more than 30 months of lees contact, consistent with the higher ethyl acetate concentrations measured in FR19 and FA19 [44].

The effects of disgorging observed here likewise mirror published findings. Both Kemp et al. (2015) [45] and Ubeda et al. (2019) [46] documented reductions in volatile esters following disgorging, typically ranging from 10–30%, a pattern reflected in the moderate but systematic decreases in ethyl acetate and isoamyl acetate between the nondisgorged and disgorged 2019 samples. Furthermore, the varietal differentiation observed in this study aligns with previous work showing that grape variety significantly influences the balance of short- and medium-chain ethyl esters, even when secondary fermentation conditions are standardized, due to inherent differences in fatty-acid metabolism and precursor availability [45,46].

#### 3.3.2. Alcohols

Higher alcohols play a complex role in wine aroma, at moderate concentrations they enhance aromatic depth, broaden the fruity and floral profile, and contribute to mouthfeel, whereas at excessive levels they may overshadow varietal notes and introduce harsh, solvent-like sensations commonly described as “fusel” attributes. International guideline issued by the OIV, acknowledge this dual impact by recommending limits that preserve wine typicity while avoiding sensory imbalance [28].

In the present dataset (Table 5), the most prominent higher alcohol across all samples is 1-pentanol, with levels spanning from 62.67 mg L^−1^ (TR19) to 106.81 mg L^−1^ (FR21d). Its relatively high abundance is noteworthy, as 1-pentanol is typically associated with green, balsamic, and slightly fruity nuances, and its concentration range aligns with values reported in studies on long-aged sparkling wines, where prolonged autolysis tends to elevate C5 and C6 alcohols.

A second compound of major relevance is 5-methyl-tetrahydrofurfuryl alcohol, a derivative often linked to Maillard-type reactions, aging processes, or wines made from botrytized fruit. Its substantial presence in the FR and TR samples, reaching 6.90 mg L^−1^ in FR19 and 6.87 mg L^−1^ in TR19d, suggests that extended maturation (36 months aging for 2019 cuvées) may have favored its formation or retention. Compounds of this structural class have been associated with caramel-like, toasted, and subtly sweet notes, consistent with findings from research on aged sparkling wines and botrytized dessert wines. By contrast, its strikingly low levels in the FA21 samples (0.61 mg L^−1^) generate a clear chemical distinction between FA and the other groups, indicating that matrix composition or shorter aging duration (9 months for 2021 wines) may strongly limit the development of furanic alcohols.

Among the remaining higher alcohols, isobutyl alcohol and 1-propanol also display meaningful variability. Isobutyl alcohol, known for its pungent and occasionally solvent-like character, appears in substantial quantities in FA21 (6.52 mg L^−1^) and FR21d (4.06 mg L^−1^) but is undetectable in FR21 and TR19d. This variability is consistent with earlier reports showing that the synthesis of branched-chain alcohols is strongly influenced by amino acid availability and nitrogen status during fermentation. Similarly, the irregular distribution of 1-propanol, which contributes subtle fruity and alcoholic notes, is notable. Its absence in TR19d, TR19, and FA21 contrasts with its presence in the other samples and may reflect differences in precursor amino acid metabolism or yeast stress adaptation, patterns also described in studies examining the influence of lees contact, base-wine nutrient composition, and fermentation temperature on higher alcohol production [47,48].

The higher-alcohol patterns observed in these sparkling wines closely mirror trends reported in traditional-method research. The consistently high levels of 1-pentanol, particularly in the long-aged FR samples, align with studies showing that extended lees aging enhances C5–C6 alcohol formation through autolytic release of fatty-acid precursors [49]. Likewise, the elevated 5-methyl-tetrahydrofurfuryl alcohol concentrations in the 2019 cuvées correspond to findings in long-matured sparkling wines and wines influenced by Maillard-type chemistry [48]. Its very low abundance in FA21 resembles the profile typically observed in younger wines with limited autolytic development. The variability recorded for isobutyl alcohol and 1-propanol echoes work demonstrating that their production is highly sensitive to nitrogen availability, amino-acid metabolism and fermentation stress [47,50,51].

#### 3.3.3. Aldehydes

The carbonyl profile of the sparkling wines offers important insight into their oxidative state and aromatic development. Aldehydes arise both from yeast metabolism during fermentation and from the oxidation of alcohols during aging, and their concentrations strongly influence freshness, complexity, and oxidative notes, an effect highlighted in previous sparkling-wine studies [52].The acetaldehyde levels observed here (54.81–89.29 mg L^−1^) fall within the range reported for traditional-method wines aged on lees, where micro-oxygenation and autolytic reactions can elevate this compound [53].

The second major aldehyde, 3-methylbutanal, varies from 0.85 to 2.65 mg L^−1^, concentrations similar to those reported in aged sparkling wines where Strecker degradation of leucine contributes malty or cocoa-like notes [54]. Propanal appears in low quantities (0.15–0.42 mg L^−1^), matching observations in Champagne and Cava where it plays a minor sensory role and forms mainly through mild oxidation of 1-propanol [55].

When comparing groups, the FR wines display the widest acetaldehyde variation and the highest 3-methylbutanal levels, indicating a more oxidative and autolysis-driven aldehyde profile. The TR wines show moderate and more uniform acetaldehyde concentrations, resembling reductively handled sparkling wines where oxygen exposure is tightly controlled [44]. The FA wines, with lower acetaldehyde but moderately high 3-methylbutanal, resemble matrices where precursor availability rather than aging length drives carbonyl formation.

#### 3.3.4. Acids

The medium-chain fatty acid (MCFA) profile reveals distinct differences across the samples and serves as a valuable indicator of yeast metabolic activity and possible sensory consequences. Yeast-derived compounds such as hexanoic, octanoic and decanoic acids can enhance complexity at low levels but tend to impart unruly or “sweaty/rancid” notes when they exceed their minimal sensory thresholds [56]. In the Fetească regală wines, these acids appear only in trace quantities, hexanoic acid under 0.11 mg/L, octanoic acid under 0.5 mg/L and decanoic acid undetectable, all well below typical perception limits, pointing to a clean, well-controlled fermentation.

In contrast, the Tămâioasă românească (TR21) and Fetească albă (FA21) wines show clearly elevated MCFA concentrations: TR21 exhibiting the highest concentrations (especially octanoic and decanoic acids) and FA21 showing increased decanoic acid. Similar upticks have been documented in white wines under yeast nutritional stress or extended autolysis, scenarios known to boost MCFA formation or release [56]. Though the measured MCFA levels in TR and FA still remain below recognized fault thresholds, they may nonetheless influence mouth-feel and contribute subtle heavier, lipid-derived aromatic nuances.

### 3.4. Sensorial Analysis

**Figure 3:**
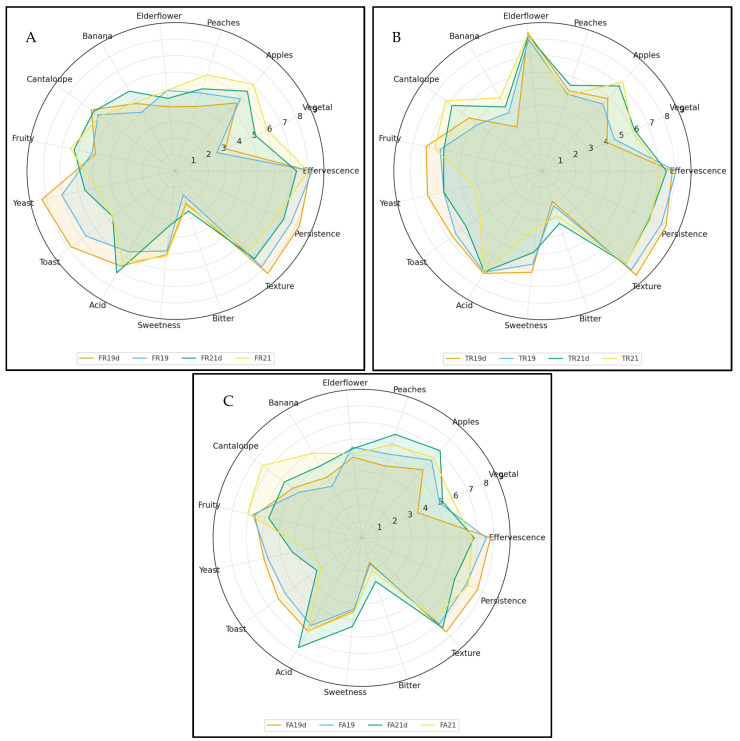
Radar plots comparing the sensory profiles of Fetească regală (**A**), Tămâioasă românească (**B**), and Fetească albă (**C**) from the 2019 and 2021 vintages, each assessed in disgorged and non-disgorged form.

The Fetească regală wines shown in Figure 3 exhibit a progressive attenuation of yeast- and toast-derived attributes from the 2019 to the 2021 vintage. This pattern is consistent with the substantial differences in maturation time on lees: the 2019 wine, with approximately 36 months of aging, develops the most pronounced autolytic character, whereas the 2021 sample, matured for only 9 months, expresses markedly weaker maturation notes. As anticipated, non-disgorged samples systematically show higher intensities of yeast and toast descriptors than their disgorged counterparts, reflecting the continued presence of suspended yeast cells and ongoing autolytic release.

In contrast, fruity descriptors such as apple, melon and banana increase in prominence across the vintages as maturation time decreases. Sensory attributes related to acidity, texture and persistence remain comparatively stable, showing minimal year-to-year variation. Taken together, these observations indicate a shift toward a fresher, more fruit-driven aromatic profile in the shorter-aged wines, with autolytic notes becoming progressively less influential.

The sensory findings align closely with the compositional data. Concentrations of ethyl hexanoate, typically associated with apple-like aromas rise steadily from 2019 to 2021, reflecting the enhanced fruit expression in the younger wines. Ethyl acetate, present at levels below 90 mg/L, contributes positively to the fruity profile without exceeding concentrations associated with sensory defects. Acetaldehyde remains below 100 mg/L and is likewise consistent with the apple-related nuances identified in the sensory assessment.

Tămâioasa românească displays a consistently pronounced floral profile, with elderflower standing out as the dominant note across all samples. While this varietal hallmark remains stable, differences emerge in the intensity of maturation-derived attributes, which diminish in wines with shorter lees-contact times, as illustrated in Figure 3. In the 2021 samples, apple, melon, and general fruity nuances are more evident, whereas in the 2019 wine these primary aromas give way to tertiary and even quaternary notes developed during extended autolysis. The analytical data support these sensory observations: ethyl acetate exceeds 60 mg/L in the degassed samples, reinforcing their fruity character, while ethyl hexanoate (apple) and ethyl caprylate (pineapple, apricot) appear at concentrations consistent with the aromatic impressions reported by the tasting panel.

The Fetească albă wines, shown in Figure 3, are marked by a fresh, fruit-driven profile, with apple, peach, melon, and banana forming the core of their aroma. Autolytic descriptors remain subdued due to the relatively short lees-contact period. Overall, the sparkling wines were perceived as lively and bright, with the high acidity typical of products obtained via the traditional Champenoise method. Ethyl acetate levels above 70 mg/L further support the fruit-forward sensory profile, aligning with the apple and peach notes frequently identified during sensory evaluation.

A higher significance of differences in sensory attributes among the samples was observed, particularly for Fetească regală and Fetească albă. Fetească regală exhibited a more complex sensory profile, characterized by pronounced fruity, floral, and toasty notes, whereas Fetească albă showed greater intensity in fruity and vegetal attributes. The detailed differences in sensory attributes among the samples are presented in Table S1 (Supplementary Materials).

**Figure 4:**
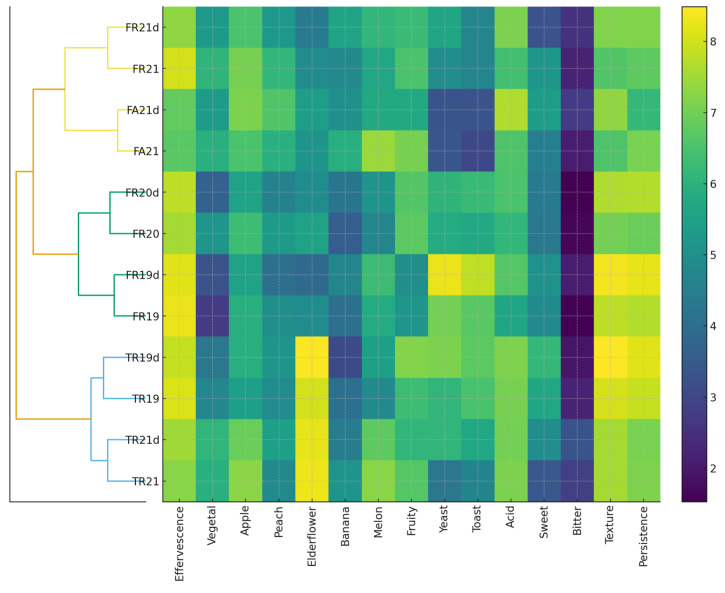
Heatmap of sensory attributes for all wine samples with hierarchical clustering applied to the samples (Ward’s method). Warmer colors indicate higher intensities across 16 sensory descriptors. The clustering reveals three main sensory groupings, distinguishing fruit-driven younger wines (FR21–FA21), floral–textural profiles (FR19–FR20), and autolytic, yeast- and toast-dominated wines (TR series).

The sensory heatmap, supported by hierarchical clustering (Figure 4), reveals clear differentiation among the wines as a function of variety, maturation time on lees, and disgorgement status. Undisgorged samples consistently show higher intensities of yeast- and toast-derived attributes, together with enhanced texture and persistence, reflecting ongoing autolytic activity from suspended yeast cells. A strong vintage effect is also evident: the 2019 wines, aged for approximately 36 months on lees, exhibit the most pronounced autolytic signatures, while the 2021 wines, with only 9 months of maturation, present a fresher aromatic profile dominated by fruit descriptors such as apple, banana, and melon. Varietal expression remains clearly distinguishable across treatments, with Fetească regală showing a balanced floral–fruity profile, TR displaying the most intense aromatic character with elevated floral and ripe-fruit notes alongside robust structural attributes, and Fetească albă expressing a more delicate and neutral style. The clustering analysis reinforces these patterns, grouping the fresher, fruit-driven FR21 and FA21 samples together; the more expressive and texturally rich FR19 wines in a second cluster; and the autolysis-dominated TR series in a third. Bitterness and sweetness remain stable across the dataset, indicating consistent vinification practices and an absence of sensory faults.

The heatmap indicates distinct patterns in the perception of acid and bitter tastes in relation to the aromatic profiles of the different samples. For the FR21 and FR21d samples, both acid and bitter intensities are moderate, which corresponds with balanced fruity notes, particularly apple and peach, and lower contributions from elderflower and banana aromas. The FA21 and FA21d samples show slightly higher acid perception but similar bitter levels, coinciding with more pronounced melon and fruity aromas, suggesting that acidity may enhance the perception of certain fruit notes. In contrast, FR20 and FR20d exhibit lower acid and bitter intensities, with weaker fruity and floral aromas, while FR19 and FR19d present moderate acid and bitter perceptions alongside stronger banana and elderflower notes, indicating a potential synergistic effect between these compounds and the bitter/acidic taste perception. The TR19 and TR19d samples display higher bitter perception coupled with moderate acidity, which aligns with enhanced elderflower intensity but subdued peach and apple notes, implying that bitterness may accentuate certain floral aromas while diminishing the perception of lighter fruit notes. Finally, TR21 and TR21d show lower acid and bitter intensities with moderate fruity and floral aromas, suggesting a more balanced profile where neither taste strongly dominates the aromatic perception. Overall, these patterns indicate that acid and bitter perceptions interact differently with specific aromatic compounds, highlighting the complexity of sensory balance in sparkling wine samples.

**Figure 5:**
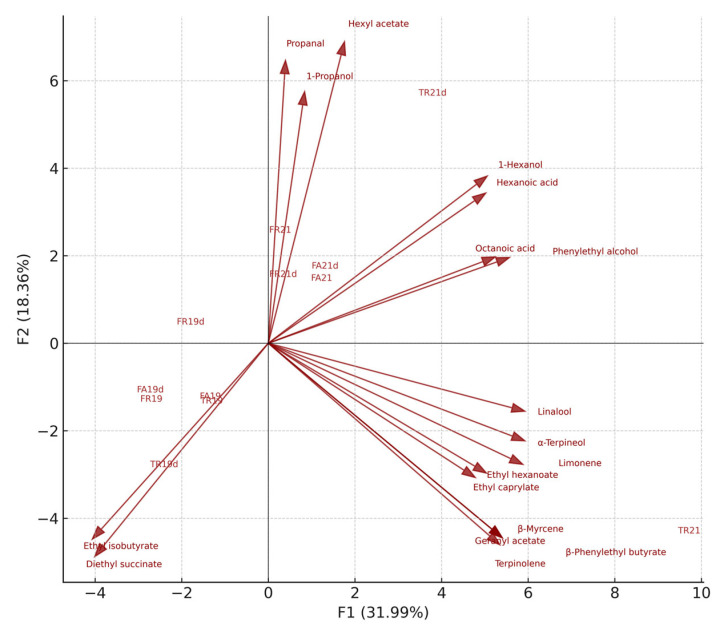
Principal component analysis (PCA) biplot (F1 × F2) showing the relationships between sparkling wine samples and their volatile aroma compounds.

The PCA biplot (Figure 5) reveals a clear separation of the sparkling wine samples along the first two principal components, which together explain nearly half of the total variance (48.66%). Samples are represented as blue bullet markers, while volatile compounds are shown as red loading vectors. Bold arrows indicate the variables with the highest contributions to PC1 and PC2, whereas faint vectors in the background represent low-contribution compounds retained for structural context. PC1 (31.43%) primarily differentiates wines based on their levels of medium-chain fatty acids, esters, and terpenes, which load strongly and positively on this axis (e.g., octanoic acid, decanoic acid, ethyl hexanoate, linalool, limonene, terpinolene). Samples positioned on the positive side of PC1, most notably TR21 and TR21d, are therefore characterized by a more intense fruity–terpenic profile and elevated concentrations of fatty-acid-derived volatiles. These compounds are commonly associated with enhanced aromatic intensity and varietal expression, particularly in Tămâioasa românească.

In contrast, wines located toward the negative PC1 space (FR19d, TR19, TR19d) are associated with higher contributions from short-chain esters, succinate derivatives, and small aldehydes, suggesting a more fermentative or autolytic aromatic signature. These loadings indicate a profile leaning toward riper fermentation notes, early autolysis markers, and less terpene-driven freshness.

PC2 (17.23%) captures variation linked to fermentative alcohols and minor aldehydes. Positive loadings include compounds such as ethyl lactate and terpineol, while negative loadings reflect higher levels of compounds such as propanal and hexyl acetate. This dimension further distinguishes wines according to the balance between fresh, green fusel alcohols versus more developed, oxidative or maturation-related volatiles. For example, TR21 scores high on PC2, suggesting elevated terpenic and maturation-driven aromas, whereas TR21d lies in the negative region of PC2, reflecting stronger fresh, alcohol contributions.

The distribution of samples also reflects varietal differences. Tămâioasa românească samples cluster mainly in the lower-right quadrant, aligning with terpene-rich vectors and confirming the variety’s strong floral–aromatic nature. Fetească regală wines occupy central to left-hand positions, consistent with more neutral fruit profiles and moderate terpene expression. Fetească albă wines appear closer to the origin or slightly toward the negative PC1 side, indicating a comparatively subtler and more fermentative volatile imprint.

## 4. Conclusions

The results of this work show that Romanian indigenous grape varieties represent a valuable and underused resource for the development of traditional-method sparkling wines. Fetească regală consistently produced base wines with high acidity and a balanced aromatic structure, demonstrating a strong ability to develop tertiary and quaternary notes during lees aging. Its volatile profile and sensory evolution indicate that this variety can support complex sparkling wine styles comparable to established international benchmarks. Tămâioasa românească, characterized by its inherently floral nature, displayed a complementary aromatic pathway, vintages with longer maturation developed a harmonious mix of floral and autolytic nuances, whereas younger wines showed a fresher, fruit-driven profile. Fetească albă, although explored to a more limited extent, showed clear promise through its clean fermentative profile and suitability for producing sparkling wines with fresh and delicate aromatics.

The chemical analyses conducted throughout the study revealed distinct groupings among the wines, emphasizing the influence of varietal origin and fermentative behavior. Variability in esters, higher alcohols, fatty acids, and terpene compounds was central to this differentiation, providing a clear chemical basis for the sensory contrasts observed. Principal component analysis strengthened these findings by identifying two dominant axes of variation: a first dimension that distinguished fruity and terpene-rich profiles from aldehydic and autolytic notes, and a second dimension reflecting differences between fusel alcohol production and the formation of fatty-acid-derived volatiles. Together, these multivariate trends highlight the interplay between grape variety, fermentation dynamics, and lees contact in shaping the aromatic identity of the resulting sparkling wines.

Overall, the study confirms that Romania’s native cultivars have both the structural acidity and aromatic versatility required for high-quality sparkling wine production by the traditional method. Their capacity to generate a wide spectrum of flavor profiles—from fresh, floral, and fruit-driven expressions to richer, autolytic styles—positions them as strong candidates for diversifying and elevating Romania’s sparkling wine sector. Continued work with these varieties may further refine their oenological potential and strengthen the regional identity of Dealu Mare within the broader landscape of European sparkling wines.

## Figures and Tables

**Table 1:** Comprehensive summary of sample codification and distinctive attributes.

Code	Grape Variety	Year	Particularity	Maturation
FR19d	Fetească regală	2019	Undisgorged	36 months
FR19	Fetească regală	2019	Disgorged	36 months
FR21d	Fetească regală	2021	Undisgorged	9 months
FR21	Fetească regală	2021	Disgorged	9 months
TR19d	Tămâioasă românească	2019	Undisgorged	36 months
TR19	Tămâioasă românească	2019	Disgorged	36 months
TR21d	Tămâioasă românească	2021	Undisgorged	9 months
TR21	Tămâioasă românească	2021	Disgorged	9 months
FA19d	Fetească albă	2019	Undisgorged	36 months
FA19	Fetească albă	2019	Disgorged	36 months
FA21d	Fetească albă	2021	Undisgorged	9 months
FA21	Fetească albă	2021	Disgorged	9 months

**Table 2:** Physico-chemical Characterization of the Produced Sparkling Wine Samples.

Sample	Alcohol (*v*/*v*)	Total Acidity (g L^−1^ Tartaric Acid)	Volatine Acidity (g L^−1^ Acetic Acid)	Free Sulfur (mg L^−1^)	Total Sulfur (mg L^−1^)	Residual Sugars (g L^−1^ )	Pressure (Bar)
FR19d	13.33 ± 0.04 ^b^	6.37 ± 0.05 ^b^	0.375 ± 0.008 ^c^	2.3 ± 0.02 ^b^	86 ± 3 ^a^	3.27 ± 0.07 ^d^	7.1 ± 0.1 ^c^
FR19	13.39 ± 0.04 ^b^	6.34 ± 0.05 ^b^	0.394 ± 0.018 ^b^	19 ± 1 ^a^	103 ± 4 ^b^	3.01 ± 0.06 ^c^	6.2 ± 0.1 ^a^
FR21d	13.13 ± 0.03 ^a^	6.48 ± 0.05 ^c^	0.375 ± 0.008 ^c^	2.1 ± 0.03 ^b^	78 ± 3 ^d^	2.15 ± 0.05 ^e^	7.4 ± 0.1 ^b^
FR21	13.06 ± 0.03 ^a^	6.53 ± 0.03 ^c^	0.394 ± 0.007 ^b^	22 ± 1.23 ^a^	112 ± 4.25 ^c^	1.95 ± 0.04 ^b^	6.8 ± 0.1 ^c^
TR19d	13.31 ± 0.04 ^b^	6.12 ± 0.02 ^a^	0.395 ± 0.008 ^b^	5.5 ± 0.12 ^d^	89 ± 3 ^a^	2.67 ± 0.06 ^a^	6.7 ± 0.1 ^d^
TR19	13.53 ± 0.05 ^c^	6.25 ± 0.01 ^a^	0.414 ± 0.013 ^a^	23 ± 0.89 ^c^	100 ± 4 ^b^	2.36 ± 0.05 ^f^	6.3 ± 0.1 ^a^
TR21d	13.42 ± 0.04 ^c^	6.24 ± 0.4 ^a^	0.365 ± 0.007 ^c^	3.5 ± 0.56 ^b^	85 ± 3 ^a^	3.67 ± 0.08 ^g^	7.4 ± 0.1 ^b^
TR21	13.38 ± 0.04 ^b^	6.29 ± 0.03 ^a^	0.384 ± 0.006 ^b^	25 ± 1.85 ^c^	117 ± 5 ^c^	3.43 ± 0.08 ^d^	6.8 ± 0.1 ^e^
FA19d	13.32 ± 0.04 ^b^	6.18 ± 0.02 ^a^	0.405 ± 0.008 ^a^	6.3 ± 0.70 ^b^	81 ± 2.50 ^a^	1.85 ± 0.04 ^b^	7.4 ± 0.1 ^b^
FA19	13.13 ± 0.03 ^a^	6.21 ± 0.01 ^a^	0.424 ± 0.004 ^a^	21 ± 1.3 ^a^	93 ± 4 ^a^	2.65 ± 0.06 ^a^	6.3 ± 0.1 ^a^
FA21d	13.29 ± 0.04 ^b^	6.28 ± 0.03 ^a^	0.405 ± 0.004 ^a^	7.3 ± 0.67 ^b^	91 ± 3 ^a^	2.85 ± 0.06 ^c^	7.4 ± 0.1 ^b^
FA21	13.13 ± 0.03 ^a^	6.31 ± 0.01 ^b^	0.424 ± 0.009 ^d^	21 ± 1.25 ^a^	105 ± 4 ^a^	2.67 ± 0.06 ^a^	6.3 ± 0.2 ^d^

Different letters indicate statistically significant differences among samples (*p* < 0.05). Values are expressed as mean ± standard deviation of three replicates. Samples sharing at least one common letter are not significantly different from each other according to Tukey’s Honest Significant Difference (HSD) test.

**Table 3:** Chromatic parameters of the analyzed samples.

Sample	Clarity (L*)	Chroma		Croma (C*)	Tonality (H)	Luminosity	Tint
		Green 0<a*>0 Red	Blue 0<b*>0 Yellow				
FR19d	98.0 ± 0.2 ^a^	−1.04 ± 0.03 ^b^	7.11 ± 0.08 ^b^	7.18 ± 0.08 ^b^	−81.71 ± 0.25 ^b^	0.14 ± 0.01 ^b^	3.87 ± 0.07 ^a^
FR19	98.0 ± 0.2 ^a^	−1.05 ± 0.03 ^b^	7.14 ± 0.08 ^b^	7.22 ± 0.07 ^b^	−81.65 ± 0.30 ^b^	0.14 ± 0.01 ^b^	3.93 ± 0.07 ^a^
FR 21d	98.7 ± 0.2 ^c^	−0.98 ± 0.02 ^b^	7.27 ± 0.08 ^c^	7.33 ± 0.08 ^c^	−82.30 ± 0.13 ^c^	0.12 ± 0.01 ^b^	5.21 ± 0.09 ^c^
FR21	98.8 ± 0.3 ^c^	−0.96 ± 0.01 ^b^	7.20 ± 0.07 ^b^	7.26 ± 0.08 ^b^	−82.41 ± 0.20 ^c^	0.12 ± 0.01 ^a^	5.23 ± 0.09 ^c^
TR19d	99.1 ± 0.2 ^d^	−0.87 ± 0.01 ^c^	5.35 ± 0.04 ^a^	5.42 ± 0.07 ^a^	−80.81 ± 0.30 ^a^	0.09 ± 0.01 ^a^	5.65 ± 10 ^d^
TR19	98.3 ± 0.1 ^b^	−0.82 ± 0.03 ^c^	6.96 ± 0.05 ^b^	7.01 ± 0.08 ^b^	−83.24 ± 0.01 ^d^	0.13 ± 0.01 ^a^	4.10 ± 0.08 ^b^
TR21d	99.1 ± 0.2 ^d^	−0.53 ± 0.02 ^d^	4.27 ± 0.0.6 ^a^	4.30 ± 0.04 ^a^	−82.89 ± 0.04 ^c^	0.08 ± 0.01 ^c^	4.81 ± 0.09 ^c^
TR21	98.2 ± 0.2 ^b^	−1.11 ± 0.03 ^a^	8.04 ± 0.07 ^d^	8.12 ± 0.09 ^d^	−82.12 ± 0.03 ^b^	0.15 ± 0.01 ^a^	4.73 ± 0.09 ^c^
FA19d	98.3 ± 0.2 ^b^	−0.97 ± 0.02 ^b^	5.9 ± 0.03 ^a^	5.98 ± 0.07 ^a^	−80.69 ± 0.03 ^a^	0.12 ± 0.01 ^a^	3.93 ± 0.06 ^a^
FA19	98.7 ± 0.2 ^c^	−0.86 ± 0.02 ^c^	6.97 ± 0.04 ^b^	7.02 ± 08 ^b^	−82.98 ± 0.02 ^c^	0.12 ± 0.01 ^a^	4.83 ± 0.08 ^c^
FA21d	98.9 ± 0.2 ^c^	−0.71 ± 0.02 ^c^	5.45 ± 0.03 ^a^	5.50 ± 0.06 ^a^	−82.59 ± 0.03 ^c^	0.09 ± 0.01 ^a^	4.85 ± 0.09 ^c^
FA21	98.6 ± 0.2 ^c^	−0.85 ± 0.02 ^c^	6.88 ± 0.05 ^b^	6.93 ± 0.06 ^b^	−82.91 ± 0.03 ^c^	0.12 ± 0.01 ^a^	4.69 ± 0.09 ^b^

Different letters indicate statistically significant differences among samples (*p* < 0.05). Values are expressed as mean ± standard deviation of three replicates. Samples sharing at least one common letter are not significantly different from each other according to Tukey’s Honest Significant Difference (HSD) test.

**Table 4:** Concentrations of Determined Esters (mg L^−1^) in the Traditional-Method Sparkling Wine Samples.

Sample	Ethyl Formate	Ethyl Acetate	Ethyl Propionate	Ethyl Isobutyrate	Ethyl Butanoate	Ethyl Isovalerate	Isoamylacetate	Ethyl Hexanoate	Hexyl Acetate	Ethyl Caprylate	Ethyl Caprate	Butanedioic Acid, Diethyl Ester
FR19d	13.97 ± 0.7	67.97 ± 14.07 *	0.59 ± 0.05	0.6 ± 0.36	1.67 ± 1.18	0.36 ± 0.05 *	3.19 ± 0.71 *	52.82 ± 11.37 *	ND	30.64 ± 7.03 *	5.36 ± 1.25 *	1.5 ± 0.13
FR19	19.67 ± 2.85 *	88.42 ± 6.8 *	0.64 ± 0.22	0.94 ± 0.32	2.91 ± 1.67	0.44 ± 0.23	4.02 ± 0.52 *	61.75 ± 1.89	ND	33.48 ± 0.45 *	4.74 ± 0.49 *	1.42 ± 0.09
FR21d	14.73 ± 4.11	72.01 ± 6.83	0.54 ± 0.08	0.61 ± 0.14	2.33 ± 1.59	0.39 ± 0.24 *	12.15 ± 0.29 *	70.53 ± 7.24 *	0.39 ± 0.24 *	69.76 ± 8.92 *	14.08 ± 1.69 *	1.19 ± 0.18
FR21	11.07 ± 1.94 *	59.98 ± 3.92 *	0.58 ± 0.16	0.71 ± 0.22	1.55 ± 0.55	0.58 ± 0.17	15.46 ± 0.8 *	59.22 ± 5.75 *	0.68 ± 0.28 *	49.1 ± 11.66 *	7.18 ± 3.65 *	0.92 ± 0.19 *
TR19d	14.77 ± 1.2	86.38 ± 6.78 *	0.44 ± 0.1	0.94 ± 0.29	3.06 ± 0.46	1.01 ± 0.08 *	2.3 ± 0.33 *	55.25 ± 3.93 *	ND	34.39 ± 31.18	7.36 ± 2.94 *	1.88 ± 0.17 *
TR19	22.71 ± 4.33 *	67.48 ± 47.58 *	0.65 ± 0.09	1.29 ± 0.16 *	2.35 ± 1.22	0.97 ± 0.26 *	5.37 ± 9.97 *	49 ± 17.17 *	ND	51.63 ± 21.66	5.77 ± 2.02 *	1.44 ± 0.38
TR21d	14.64 ± 2.49	61.11 ± 10.3 *	0.48 ± 0.18	0.62 ± 0.34	0.99 ± 0.07	0.67 ± 0.03	10.98 ± 0.46 *	58.04 ± 12.27 *	2.25 ± 0.48 *	41.48 ± 8.38 *	4.34 ± 0.49 *	0.93 ± 0.13 *
TR21	14.01 ± 2.58	70.72 ± 1.73	0.45 ± 0.04	0.5 ± 0.06 *	1.63 ± 0.81	0.63 ± 0.13	9.98 ± 0.37 *	80.64 ± 2.32 *	2.91 *	93.03 ± 13.23 *	6.41 ± 1.07 *	1.04 ± 0.08 *
FA19d	14.64 ± 2.49	61.11 ± 10.3 *	0.48 ± 0.18	0.62 ± 0.34	0.99 ± 0.07	0.67 ± 0.03	10.98 ± 0.46 *	58.04 ± 12.27 *	2.25 ± 0.48 *	41.48 ± 8.38 *	4.34 ± 0.49 *	0.93 ± 0.13 *
FA19	17.7 ± 0.06	87.33 ± 8.01 *	0.62 ± 0.06	1.27 ± 0.12 *	3.89 ± 1.59	0.61 ± 0.28 *	13.89 ± 1.04 *	57.89 ± 3.36 *	0.34 ± 0.13 *	35.31 ± 4.22 *	7.16 ± 1.63 *	1.44 ± 0.23
FA21d	16.26 ± 2.32	83.55 ± 7.09 *	0.63 ± 0.15	1.2 ± 0.33 *	2.38 ± 1.45	0.83 ± 0.15 *	13.24 ± 2.37 *	62.27 ± 5.42	0.44 ± 0.11 *	58.71 ± 24.67	8.29 ± 1.76 *	1.38 ± 0.1
FA21	12.44 ± 1.12	71.23 ± 4.58	0.59 ± 0.2	0.66 ± 0.28	3.68 ± 0.35	0.8 ± 0.07	9.53 ± 0.58 *	66.92 ± 4.3 *	0.46 ± 0.11 *	90.35 ± 7.64 *	6.49 ± 0.56 *	1.04 ± 0.15

* indicate statistically significant differences among samples (*p* < 0.05), based on one-way ANOVA followed by Tukey’s HSD post hoc test. ND values were treated as zero. ND—not detectable. All the values are expressed in mg L^−1^.

**Table 5:** Volatile compound composition (higher alcohols, aldehydes, and fatty acids) of the sparkling wines.

Sample	FR19d	FR19	FR21d	FR21	TR19d	TR19	TR21d	TR21	FA19d	FA19	FA21d	FA21
Higher alcohols												
Propanol	1.59 ± 0.05 *	2.26 ± 0 *	2.44 ± 0.14 *	2.68 ± 0.73 *	ND	ND	2.29 ± 0.43 *	2.02 ± 0 *	2.66 ± 0 *	2.19 ± 0.84 *	2.29 ± 0.22 *	ND
Isobutil alcohol	5.37 ± 5.99 *	1.74 ± 0.64 *	4.06 ± 0.39 *	ND	ND	2.7 ± 0 *	1.79 ± 0 *	1.59 ± 0 *	1.26 ± 0.13	1.41 ± 0.08	5.49 ± 3.44	6.52 ± 1.32
MTHFA **	4.52 ± 0.87	6.9 ± 3.3	4.75 ± 0	5.71 ± 3.76	6.87 ± 2.01	6.83 ± 3.67	5.74 ± 2.84	5.35 ± 0.97	6.43 ± 5.32	6.3 ± 2.81	0.61 ± 0 *	ND
3-hexanol	0.09 ± 0	0.09 ± 0.02	0.12 ± 0.03	0.08 ± 0.01	0.11 ± 0.02	ND	ND	0.2 ± 0.12	0.13 ± 0.01	0.11 ± 0.05	0.21 ± 0.07	0.15 ± 0.08
1-pentanol	73 ± 2.09 *	78.87 ± 6.82 *	106.81 ± 14.01 *	91.92 ± 9.64 *	77.9 ± 4.19 *	62.67 ± 26.19 *	95.87 ± 8.37 *	101.25 ± 4.29 *	91.89 ± 1.22 *	91.81 ± 6.81 *	99.28 ± 7.23 *	87.51 ± 3.18 *
1-hexanol	0.63 ± 0.07 *	0.47 ± 0.07 *	0.94 ± 0.27 *	0.87 ± 0.18 *	0.92 ± 0.02 *	0.71 ± 0.24 *	2.21 ± 0.38 *	1.99 ± 0.12 *	0.5 ± 0.21 *	0.48 ± 0.14 *	1.28 ± 0.15 *	1.1 ± 0.08 *
Phenylethyl alcohol	0.22 ± 0.02 *	0.16 ± 0.03 *	0.28 ± 0.02 *	0.2 ± 0.01 *	0.23 ± 0.01 *	0.23 ± 0.05 *	0.46 ± 0.09 *	0.45 ± 0.02 *	0.23 ± 0.06 *	0.23 ± 0.01 *	0.44 ± 0.07 *	0.32 ± 0.08 *
Aldehydes												
Acetaldehyde	82.22 ± 4.06 *	88.05 ± 12.93 *	89.29 ± 2.16 *	83.11 ± 11.67	74.09 ± 15.05 *	54.81 ± 9.82 *	79.3 ± 8.2 *	64.99 ± 24.18 *	69.38 ± 10.12 *	73.16 ± 6.11 *	67.94 ± 7.65 *	64.33 ± 4.81 *
Propanal	0.15 ± 0.07	0.27 ± 0.2	0.4 ± 0.26 *	0.42 ± 0.17 *	0.27 ± 0.19	0.26 ± 0.11	0.26 ± 0.11	0.22 ± 0.2	0.42 ± 0.06 *	0.23 ± 0.09	0.24 ± 0.14	0.21 ± 0.07
3-methylbutanal	0.85 ± 1.04 *	1.16 ± 1.72	2.5 ± 0.14 *	2.23 ± 0.52 *	2.65 ± 0.33 *	2.13 ± 0.48 *	2.36 ± 0.12 *	0.92 ± 0	1.07 ± 0.86	0.93 ± 0.15	2.09 ± 1.18 *	2.41 ± 0.18 *
Acids												
Hexanoic acid	0.05 *	ND	ND	0.08 ± 0.03 *	0.09 ± 0.01 *	0.11 ± 0 *	0.1 ± 0 *	0.11 ± 0.04 *	0.29 ± 0.04 *	0.27 ± 0 *	0.2 ± 0.03 *	0.13 ± 0.01 *
Octanoic Acid	0.19 ± 0.02 *	0.1 ± 0.04 *	0.05 ± 0 *	0.06 ± 0.05 *	0.39 ± 0.08 *	0.45 ± 0.03 *	0.6 ± 0.07 *	0.61 ± 0.18 *	1.16 ± 0.44 *	1.21 ± 0.14 *	0.99 ± 0.18 *	0.8 ± 0.15 *
Decanoic acid	ND	ND	ND	ND	ND	ND	0.18 ± 0.01	ND	0.43 ± 0.05	0.61 ± 0.18	0.53 ± 0.36	0.34 ± 0.08

* indicate statistically significant differences among samples (*p* < 0.05), based on one-way ANOVA followed by Tukey’s HSD post hoc test. ND values were treated as zero. ND—not detectable. All the values are expressed in mg L^−1^; ** MTHFA—5-methyl-tetrahydrofurfuryl alcohol.

## Data Availability

The original contributions presented in the study are included in the article/supplementary material, further inquiries can be directed to the corresponding author.

## Supplementary Materials

The following supporting information can be downloaded at https://www.mdpi.com/article/10.3390/foods15020353/s1: Table S1: Significance of differences in sensory attributes.

## Abbreviations

The following abbreviations are used in this manuscript:

FA	Fetească albă
FR	Fetească regală
TR	Tămâioasă românească
AF	Alcoholic fermentation
OIV	International Organization of Vine and Wine
SO_2_	Sulfur dioxide
CO_2_	Carbon dioxide
MTHFA	5-methyl-tetrahydrofurfuryl alcohol
MCFA	Medium-chain fatty acid

## References

[1] Luchian C.E., Grosaru D., Scutarașu E.C., Colibaba L.C., Scutarașu A., Cotea V.V. (2025). Advancing Sparkling Wine in the 21st Century: From Traditional Methods to Modern Innovations and Market Trends. Fermentation.

[2] Tudela R., Gallardo-Chacon J.J., Rius N., Lopez-Tamames E., Buxaderas S. (2012). Ultrastructural changes of sparkling wine lees during long-term aging in real enological conditions. FEMS Yeast Res..

[3] Jové P., Mateu-Figueras G., Bustillos J., Martín-Fernández J.A. (2024). Analysis of Aromatic Fraction of Sparkling Wine Manufactured by Second Fermentation and Aging in Bottles Using Different Types of Closures. Processes.

[4] Buxaderas S., López-Tamames E., Henry J. (2012). Chapter 1—Sparkling Wines: Features and Trends from Tradition. Advances in Food and Nutrition Research.

[5] Mihaela Dana Pop A. (2024). The Influence of Physical-Chemical Parameters on the Sensory Evaluation of Sparkling Wines. Ann. ‘Valahia’ Univ. Târgovişte Agric..

[6] Martínez-Rodríguez A.J., Carrascosa A.V., Martín-Álvarez P.J., Moreno-Arribas V., Polo M.C. (2002). Influence of the yeast strain on the changes of the amino acids, peptides and proteins during sparkling wine production by the traditional method. J. Ind. Microbiol. Biotechnol..

[7] Tofalo R., Perpetuini G., Rossetti A.P., Gaggiotti S., Piva A., Olivastri L., Cichelli A., Compagnone D., Arfelli G. (2022). Impact of Saccharomyces cerevisiae and non-Saccharomyces yeasts to improve traditional sparkling wines production. Food Microbiol..

[8] Martínez-García R., García-Martínez T., Puig-Pujol A., Mauricio J.C., Moreno J. (2017). Changes in sparkling wine aroma during the second fermentation under CO_2_ pressure in sealed bottle. Food Chem..

[9] Nunez Y.P., Carrascosa A.V., González R., Polo M.C., Martínez-Rodríguez A.J. (2005). Effect of accelerated autolysis of yeast on the composition and foaming properties of sparkling wines elaborated by a champenoise method. J. Agric. Food Chem..

[10] Coldea T.E., Mudura E., Fărcaș A., Marc L. (2016). Valorisation of hybrid grape variety into processing of red sparkling wine. J. Agroaliment. Process. Technol..

[11] Focea M., Luchian C.-E., Zamfir C.-I., Niculaua M., Moroșanu A.-M., Nistor A.-M., Andrieș M.-T., Lăcureanu F.-G., Cotea V.V. (2017). Organoleptic Chracteristics of Experimental Sparkling Wines. https://repository.iuls.ro/st/web/viewer.html?file=https://repository.iuls.ro/xmlui/bitstream/handle/20.500.12811/991/LSH_v.60_nr.2_Organoleptic%20chracteristics%20of....pdf?sequence=1&isAllowed=y.

[12] Cotan Ș.-D., Popîrdă A., Luchian C.-E., Colibaba L.-C., Zamfir C.-I., Cotea V.V., Nistor A.-M. (2020). Study of Some Sparkling Wines Obtained from Local Varieties of White Grapes in the Cotnari Vineyard. https://repository.iuls.ro/st/web/viewer.html?file=https://repository.iuls.ro/xmlui/bitstream/handle/20.500.12811/4531/HOR-TI_v.63_nr.2_Study%20of%20some%20sparkling%20wines%20obtained%20from%20local%20varieties%20of%20white%20grapes%20...pdf?sequence=1&isAllowed=y.

[13] Donici A., Oslobanu A., Fitiu A., Babes A.C., Bunea C.I. (2016). Qualitative assessment of the white wine varieties grown in Dealu Bujorului vineyard, Romania. Not. Bot. Horti Agrobot. Cluj-Napoca.

[14] Bedreag I.C., Cioroiu I.-B., Niculaua M., Nechita C.-B., Cotea V.V. (2025). Volatile Compounds as Markers of Terroir and Winemaking Practices in Fetească Albă Wines of Romania. Beverages.

[15] Irimia L.M., Patriche C.V., Quénol H. (2014). Analysis of viticultural potential and delineation of homogeneous viticultural zones in a temperate climate region of Romania. OENO One.

[16] Chircu C., Muste S., Birou A.-M., Man S. (2011). Comparative data regarding the grapes maturation evolution at Feteasca Regala, Chardonnay and Pinot Noir varieties during 2008–2010, in the Jidvei vineyard. Bull. Univ. Agric. Sci. Veter-Med. Cluj-Napoca Agric..

[17] Zaldea G., Damian D., Pârcălabu L., Iliescu M., Enache V., Tănase A., Bosoi I., Ghiur A. (2022). The Evolution of Climatic Conditions Between 1989 and 2021 in Representative Vine areas of Romania. https://repository.iuls.ro/st/web/viewer.html?file=https://repository.iuls.ro/xmlui/bitstream/handle/20.500.12811/4710/HOR-TI_v.65_nr.2_The%20evolution%20of%20climatic%20conditions%20between%201989%20and%202021.pdf?sequence=1&isAllowed=y.

[18] Vizitiu D.E., Buciumeanu E.-C., Dincă L., Radomir A.-M. (2022). Contributions to a durable Viticulture in Dealu Mare Vineyard with the Analytic Hierarchy Process. In *Scientific Studies & Research. Series Biology/Studii si Cercetari Stiintifice. Seria Biologie*. https://www.researchgate.net/profile/Radomir-Ana-Maria/publication/368830856_CONTRIBUTIONS_TO_A_DURABLE_VITICULTURE_IN_DEALU_MARE_VINEYARD_WITH_THE_ANALYTIC_HIERARCHY_PROCESS/links/63fc5b59b1704f343f868d6e/CONTRIBUTIONS-TO-A-DURABLE-VITICULTURE-IN-DEALU-MARE-VINEYARD-WITH-THE-ANALYTIC-HIERARCHY-PROCESS.pdf.

[19] OIV (2025). Compendium of International Methods of Wine and Must Analysis.

[20] Dumitriu G.-D., Teodosiu C., Gabur I., Cotea V.V., Peinado R.A., López de Lerma N. (2021). Alternative Winemaking Techniques to Improve the Content of Phenolic and Aromatic Compounds in Wines. Agriculture.

[21] Odăgeriu G.C.S., Cotea V.V., Bârliga N., Ciubucă A. (2000). Caracteristicile Cromatice C. I. E. Lab-76 Ale Vinurilor Roşii din Podgoria Bujoru. “Lucrări ştiinţifice”.

[22] Sauciuc J., Odǎgeriu G., Tudose I., Negurǎ C. (1997). Caracteristicile cromatice, C.I.E. Lab 76, alevinurilor din podgoria Copou Iaşi.

[23] OIV Review Document on Sensory Analysis of Wine. https://www.oiv.int/public/medias/3307/review-on-sensory-analysis-of-wine.pdf.

[24] Just-Borràs A., Moroz E., Giménez P., Gombau J., Ribé E., Collado A., Cabanillas P., Marangon M., Fort F., Canals J.M. (2024). Comparison of ancestral and traditional methods for elaborating sparkling wines. Curr. Res. Food Sci..

[25] Raymond Eder M.L., Rosa A.L. (2021). Non-Conventional Grape Varieties and Yeast Starters for First and Second Fermentation in Sparkling Wine Production Using the Traditional Method. Fermentation.

[26] Cisilotto B., Scariot F.J., Schwarz L.V., Mattos Rocha R.K., Longaray Delamare A.P., Echeverrigaray S. (2023). Are the characteristics of sparkling wines obtained by the Traditional or Charmat methods quite different from each other?. OENO One.

[27] Pîrcălabu L., Barbu S.P., Marian, Ion Tudor G., Bucur A. (2025). Evaluation of the Technological Potential of Wine Grape Varieties in the Context of Climate Change in the Dealu Mare Vineyard. https://horticulturejournal.usamv.ro/pdf/2025/issue_1/Art41.pdf.

[28] OIV International Code of Oenological Practices. https://www.oiv.int/sites/default/files/publication/2025-04/CPO%202025%20EN.pdf.

[29] Charnock H., Pickering G., Kemp B. (2023). The impact of dosage sugar-type and aging on Maillard reaction-associated products in traditional method sparkling wines. OENO One.

[30] Cotea V.V., Focea M.C., Luchian C.E., Colibaba L.C., Scutarasu E.C., Marius N., Zamfir C.I., Popirda A. (2021). Influence of Different Commercial Yeasts on Volatile Fraction of Sparkling Wines. Foods.

[31] Aliev T., Korolev I., Yasnov M., Nosonovsky M., Skorb E.V. (2025). Rose or white, glass or plastic: Computer vision and machine learning study of cavitation bubbles in sparkling wines. RSC Adv..

[32] Liger-Belair G. (2005). The physics and chemistry behind the bubbling properties of champagne and sparkling wines: A state-of-the-art review. J. Agric. Food Chem..

[33] Miliordos D.E., Kontoudakis N., Kouki A., Kanapitsas A., Lola D., Goulioti E., Kotseridis Y. (2025). Influence of vintage and grape maturity on volatile composition and foaming properties of sparkling wine from Savvatiano (*Vitis vinifera* L.) variety. OENO One.

[34] Just-Borràs A., Alday-Hernández M., García-Roldán A., Bustamante M., Gombau J., Cabanillas P., Rozès N., Canals J.M., Zamora F. (2024). Assessment of Physicochemical and Sensory Characteristics of Commercial Sparkling Wines Obtained Through Ancestral and Traditional Methods. Beverages.

[35] Scutarasu E.C., Luchian C.E., Vlase L., Colibaba L.C., Gheldiu A.M., Cotea V.V. (2021). Evolution of phenolic profile of white wines treated with enzymes. Food Chem..

[36] Buican B.-C., Colibaba L.C., Luchian C.E., Kallithraka S., Cotea V.V. (2023). “Orange” Wine—The Resurgence of an Ancient Winemaking Technique: A Review. Agriculture.

[37] Hensel M., Di Nonno S., Mayer Y., Scheiermann M., Fahrer J., Durner D., Ulber R. (2022). Specification and Simplification of Analytical Methods to Determine Wine Color. Processes.

[38] Gil-Muñoz R., Gómez-Plaza E., Martínez A., López-Roca J.M. (1997). Evolution of the CIELAB and other spectrophotometric parameters during wine fermentation. Influence of some pre and postfermentative factors. Food Res. Int..

[39] He Y., Wang X., Li P., Lv Y., Nan H., Wen L., Wang Z. (2023). Research progress of wine aroma components: A critical review. Food Chem..

[40] Ribéreau-Gayon P., Dubourdieu D., Donèche B., Lonvaud A. (2006). Handbook of Enology, Volume 1: The Microbiology of Wine and Vinifications.

[41] Swiegers J.H., Bartowsky E.J., Henschke P.A., Pretorius I.S. (2005). Yeast and bacterial modulation of wine aroma and flavour. Aust. J. Grape Wine Res..

[42] Pinheiro S.S., Campos F., Cabrita M.J., Silva M.G.D. (2025). Exploring the Aroma Profile of Traditional Sparkling Wines: A Review on Yeast Selection in Second Fermentation, Aging, Closures, and Analytical Strategies. Molecules.

[43] Riu-Aumatell M., Bosch-Fusté J., López-Tamames E., Buxaderas S. (2006). Development of volatile compounds of cava (Spanish sparkling wine) during long ageing time in contact with lees. Food Chem..

[44] Martinez-Garcia R., Mauricio J.C., Garcia-Martinez T., Peinado R.A., Moreno J. (2021). Towards a better understanding of the evolution of odour-active compounds and the aroma perception of sparkling wines during ageing. Food Chem..

[45] Kemp B., Alexandre H., Robillard B., Marchal R. (2015). Effect of production phase on bottle-fermented sparkling wine quality. J. Agric. Food Chem..

[46] Ubeda C., Kania-Zelada I., Del Barrio-Galan R., Medel-Maraboli M., Gil M., Pena-Neira A. (2019). Study of the changes in volatile compounds, aroma and sensory attributes during the production process of sparkling wine by traditional method. Food Res. Int..

[47] Liu S., Ma D., Li Z., Sun H., Mao J., Shi Y., Han X., Zhou Z., Mao J. (2021). Assimilable nitrogen reduces the higher alcohols content of huangjiu. Food Control.

[48] Carrau F.M., Medina K., Farina L., Boido E., Henschke P.A., Dellacassa E. (2008). Production of fermentation aroma compounds by Saccharomyces cerevisiae wine yeasts: Effects of yeast assimilable nitrogen on two model strains. FEMS Yeast Res..

[49] Sawyer S., Longo R., Solomon M., Nicolotti L., Westmore H., Merry A., Gnoinski G., Ylia A., Dambergs R., Kerslake F. (2021). Autolysis and the duration of ageing on lees independently influence the aroma composition of traditional method sparkling wine. Aust. J. Grape Wine Res..

[50] Christofi S., Papanikolaou S., Dimopoulou M., Terpou A., Cioroiu I.B., Cotea V., Kallithraka S. (2022). Effect of Yeast Assimilable Nitrogen Content on Fermentation Kinetics, Wine Chemical Composition and Sensory Character in the Production of Assyrtiko Wines. Appl. Sci..

[51] Li J., Yuan M., Meng N., Li H., Sun J., Sun B. (2024). Influence of nitrogen status on fermentation performances of non--Saccharomyces yeasts: A review. Food Sci. Hum. Wellness.

[52] Bueno M., Marrufo-Curtido A., Carrascon V., Fernandez-Zurbano P., Escudero A., Ferreira V. (2018). Formation and Accumulation of Acetaldehyde and Strecker Aldehydes during Red Wine Oxidation. Front. Chem..

[53] Cejudo-Bastante M.J., Hermosín-Gutiérrez I., Pérez-Coello M.S. (2011). Micro-oxygenation and oak chip treatments of red wines: Effects on colour-related phenolics, volatile composition and sensory characteristics. Part I: Petit Verdot wines. Food Chem..

[54] Marrufo-Curtido A., de-la-Fuente-Blanco A., Saenz-Navajas M.P., Ferreira V., Bueno M., Escudero A. (2021). Sensory Relevance of Strecker Aldehydes in Wines. Preliminary Studies of Its Removal with Different Type of Resins. Foods.

[55] Boschfuste J., Riuaumatell M., Guadayol J., Caixach J., Lopeztamames E., Buxaderas S. (2007). Volatile profiles of sparkling wines obtained by three extraction methods and gas chromatography–mass spectrometry (GC–MS) analysis. Food Chem..

[56] Csutoras C., Bakos-Barczi N., Burkus B. (2022). Medium chain fatty acids and fatty acid esters as potential markers of alcoholic fermentation of white wines. Acta Aliment..

