# Supplementary material for: From Vine to Sparkle: An Analytical and Sensory Evaluation of Sparkling Wines from Some Romanian Native Grapes
Source: Foods. 2026 Jan 18;15(2):353. doi: 10.3390/foods15020353 (PMC12840789; doi:10.3390/foods15020353)
Supplement: Supplementary file 1 [file foods-15-00353-s001.zip › foods-4065268-supplementary.pdf]

Table S1. Significance of differences in sensory attributes among the samples

Sample	Effervscence	Vegetal	Apple	Peach	Elderflower	Banana	Melon	Fruity	Yeast	Toast	Acid	Sweet	Bitter	Texture	Persistence
**FR19d**	-	*	-	*	*	*	-	*	*	*	*	*	-	*	*
**FR19**	-	-	-	-	-	-	-	-	-	-	-	-	-	-	-
**FR21d**	*	-	-	-	-	*	-	-	-	-	-	-	-	*	*
**FR21**	*	-	-	*	-	-	-	-	-	-	-	-	-	*	*
**TR19d**	-	-	-	-	*	*	-	*	-	-	-	-	-	-	-
**TR19**	*	-	-	-	*	-	-	*	-	-	-	-	-	-	*
**TR21d**	-	-	-	-	-	-	-	-	-	-	-	-	-	-	-
**TR21**	-	-	-	-	-	-	-	-	-	-	-	-	-	-	-
**FA19d**	-	*	*	*	*	*	-	-	-	-	*	-	-	*	*
**FA19**	-	-	*	*	-	*	-	-	-	-	*	-	-	*	*
**FA21d**	*	*	-	-	-	-	-	*	-	-	*	-	-	-	*
**FA21**	-	*	-	-	-	-	-	*	-	-	-	-	-	*	*

*indicate statistically significant differences among samples ($p < 0.05$), based on one-way ANOVA followed by Tukey’s HSD post-hoc test. “–“ indicate no statistically significant difference
